# Mutational landscape of HSP family on human breast cancer

**DOI:** 10.1038/s41598-024-61807-8

**Published:** 2024-05-30

**Authors:** Juan Manuel Fernandez-Muñoz, Martin Eduardo Guerrero-Gimenez, Leonardo Andrés Ciocca, María José Germanó, Felipe Carlos Martin Zoppino

**Affiliations:** 1 Laboratory of Data Science and Genomics, IMBECU CONICET UNCuyo, 5500 Mendoza, Argentina; 2 Medicine School, National University of Cuyo, 5500 Mendoza, Argentina; 3 Hospital Italiano de Mendoza, Mendoza, 5519 Argentina

**Keywords:** Breast cancer, Cancer genomics, Breast cancer

## Abstract

Breast cancer (BRCA) is a prevalent malignancy with the highest incidence among females. BRCA can be categorized into five intrinsic molecular subtypes (LumA, LumB, HER2, Basal, and Normal), each characterized by varying molecular and clinical features determined by the expression of intrinsic genes (PAM50). The Heat Shock Protein (HSP) family is composed of 95 genes evolutionary conservated, they have critical roles in proteostasis in both normal and cancerous processes. Many studies have linked HSP to the development and spread of cancer. They modulate the activity of multiple proteins expressed by oncogenes and anti-oncogenes through a range of interactions. In this study, we evaluate the mutational changes that HSP undergoes in BRCA mainly from the TCGA database. We observe that Copy Number Variations (CNV) are the more frequent events analyzed surpassing the occurrence of point mutations, indels, and translation start site mutations. The Basal subtype showcased the highest count of amplified CNV, including subtype-specific changes, whereas the Luminals tumors accumulated the greatest number of deletion CNV. Meanwhile, the HER2 subtype exhibited a comparatively lower frequency of CNV alterations when compared to the other subtypes. This study integrates CNV and expression data, finding associations between these two variables and the influence of CNV on the deregulation of HSP expression. To enhance the role of HSP as a risk predictor in BRCA, we succeeded in identifying CNV profiles as a prognostic marker. We included Artificial Intelligence to improve the clustering of patients, and we achieved a molecular CNV signature as a significant risk factor independent of known classic markers, including molecular subtypes PAM50. This research enhances the comprehension of HSP DNA alterations in BRCA and its relation with predicting the risk of affected individuals providing insights to develop guide personalized treatment strategies.

## Introduction

Breast cancer (BRCA) is a global public health issue highlighted by age-standardized incidence and mortality rates of 47.8 and 13.6, respectively^1^. According to molecular aspects, BRCA can be divided into five intrinsic subtypes (Luminal A, Luminal B, Basal, HER2, and Normal-like), each exhibiting distinct clinical characteristics. The potential of this classification method to stratify breast cancer cases and enable tailored treatment strategies makes it a vital tool in clinical practice^2,3^. The Heat Shock Protein family (HSP) is composed of 95 genes subdivided into five subfamilies^4^, these genes play critical roles in proteostasis in both normal and cancerous states. Many members of the HSP family have been extensively studied on relevant pathways of cancer hallmarks^5,6^. We have previously described the deregulation of various HSP in BRCA, identifying both up-regulated genes and others that are strongly repressed. We demonstrated that multiple members of the HSP family are closely associated with patient survival and are expressed in characteristic patterns, enabling the identification of three subtypes of HSP patients that are also associated with patient outcomes^7^. Genetic variations in the human genome encompass a broad range of alterations, spanning from large-scale chromosomal anomalies (loss of chromosomes or segmental aneuploidy) to small-scale single nucleotide variants (SNP)^8^. From this vantage point, extensive research has been dedicated to investigating DNA alterations that have the potential to impact on tumoral process. In pursuit of this objective, SNP have emerged as the most extensively studied mutations, revealing associations with various types of cancer as well as other genetic disorders. As we embrace advanced techniques such as microarrays and next-generation sequencing, our understanding of genome-wide alterations has expanded significantly. Among the noteworthy alterations that have come to light, copy number variations (CNV) stand out as a particularly relevant and consequential aspect of the genomic landscape in the context of cancer^9,10^. CNV refers to changes in the number of copies of a particular DNA region ranging from 1 kilobase (1 Kb) to 5 megabases (5 Mb) in length. These changes can result in either gains or losses of DNA within the genome and can have significant effects on gene expression^11,12^. The exploration and comprehension of CNV within the context of BRCA have not only yielded indispensable insights into the disease's underlying mechanisms but have also assumed critical relevance in its diagnosis, prognosis, and treatment^13^. A prominent illustration of this impact can be observed in BRCA, particularly concerning the ERBB2 (HER2) driver gene. This gene is frequently activated through an increase in its copy number, a phenomenon found in roughly 15–20% of BRCA cases, often associated with an aggressive subtype of the disease. The overexpression of HER2 instigates unbridled cancer cell proliferation, rendering it a crucial therapeutic target for the development of specific treatments against this aberration^14^. In this study, we performed an integral analysis of alterations in all genes of the HSP family in BRCA, focusing on CNV, on the TCGA and METABRIC cohorts, taking into consideration the molecular subtypes of BRCA and incorporating transcriptomic and clinical data.

## Results

### Point mutations on HSP genes

**Figure 1:**
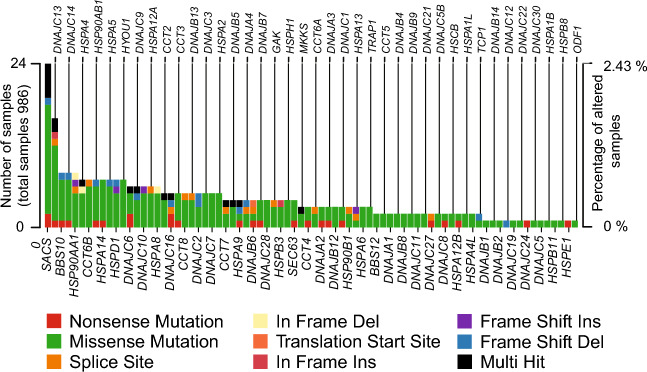
The landscape of HSP alterations in human breast cancer (TCGA cohort): point mutations, indels, and modifications at the translation start site. The mutation frequency of each HSP gene is remarked.

BRCA is a complex disease where active participation of the HSP family members in cellular processes related to cancer has been reported. We started this work evaluating alterations in the DNA of HSP genes, in the first instance we studied point mutations (nonsense, missense, splice sites mutations), indels (in frame or frameshift), translation start site mutations, and multi-hit cases (a gene that has been point-mutated multiple times within a single tumor sample). We determined that these types of DNA alterations are presented across 78 HSP genes, and occur at low frequency in the cohort, reaching a maximum occurrence of 2.43% found in the region of the SACS gene. Over the alterations studied until here, we observed a prevalence of missense mutations over the other types (p = 2.418e−12, proportional Z-test) (Fig. 1). Next, we analyzed the mutation frequency of HSP genes according to their length and we compared them against the mutation frequency of total mutated genes (16,041). We identified 73 HSP with a significant difference in mutation frequency. Of these, 31 exhibited a higher mutation frequency, while 42 showed a lower frequency (Fig. S1, and Table S1).

**Figure 2:**
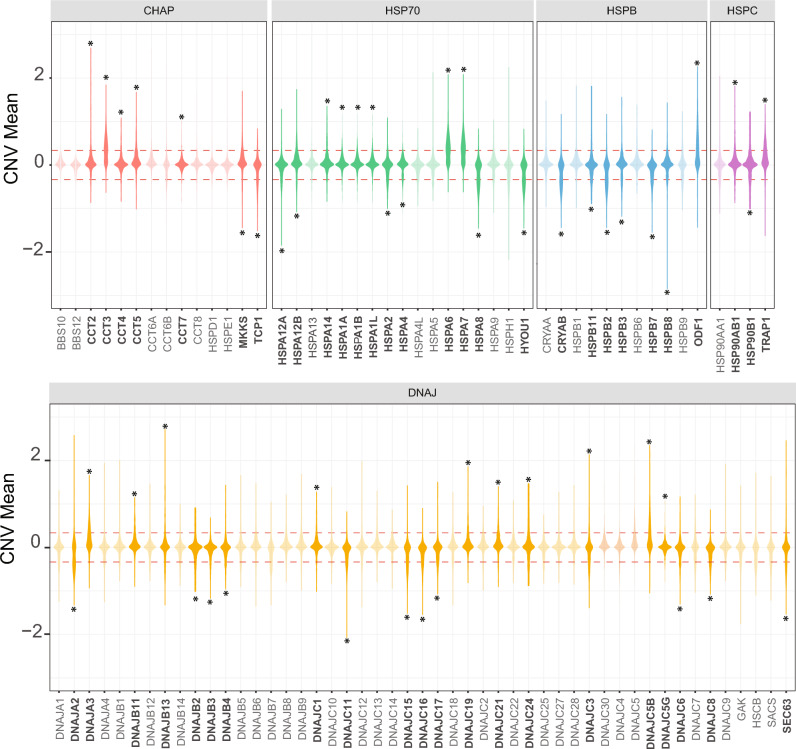
The landscape of HSP CNV of human breast cancer ordered by subfamilies (TCGA cohort n = 1094). Violin plot of CNV values of HSP genes. The punctate red line shows the cut-off (amp-CNV ≥ 0.3 and del-CNV ≤ − 0.3) to CNV alteration. The asterisk indicates recurrent significative amp-CNV or del-CNV obtained by GAIA.

Following this, we conducted a complete analysis of CNV in HSP genes within the entire BRCA TCGA cohort, with a specific emphasis on each HSP subfamily. As an initial step, we assessed the distribution and relative proportion of CNV within each of these genes. Subsequently, to find recurrent and statistically significant CNV of genome regions we used GAIA package^15^. We found 24 amplified CNV (amp-CNV) genes and 26 copy-number loss (del-CNV) genes (Fig. 2). The CHAP subfamily includes genes that encoded CCT complex (chaperonin containing TCP-1) that folds nearly 10% of the proteome^16^. In this subfamily, we identified genes with amp-CNV alterations in CCT2, CCT3, CCT4, CCT5, and CCT7 meanwhile, TCP1 presented del-CNV. Another gene found with del-CNV is MKKS which is involved in BBsome and in the formation of centrosomes and ciliums^17,18^. The subfamily HSP70 presented del-CNV in several members (HSPA12A, HSPA12B, HSPA2, HSPA4, HSPA8, and HYOU1), amp-CNV were detected in six genes (HSPA14, HSPA1A, HSPA1B, HSPA1L, HSPA6, and HSPA7). The subfamily of small HSP known as HSPB showed a preponderance of del-CNV (CRYAB, HSPB2, HSPB3, HSPB7, HSPB11, and HSPB8) with just a divergent member with amp-CNV (ODF1). One of the most studied subfamilies in cancer, the HSPC subfamily (also known as HSP90) presented 2 amp-CNV altered members (HSP90AB1, and TRAP1), and just one del-CNV (HSP90B1). The vast DNAJ subfamily showed a mixture of del-CNV (DNAJA2, DNAJB2, DNAJB3, DNAJB4, DNAJC11, DNAJC15, DNAJC16, DNAJC17, DNAJC6, DNAJC8, and SEC63) and amp-CNV (DNAJA3, DNAJB11, DNAJB13, DNAJC1, DNAJC19, DNAJC21, DNAJC24, DNAJC3, DNAJC5B and DNAJC5G).

**Figure 3:**
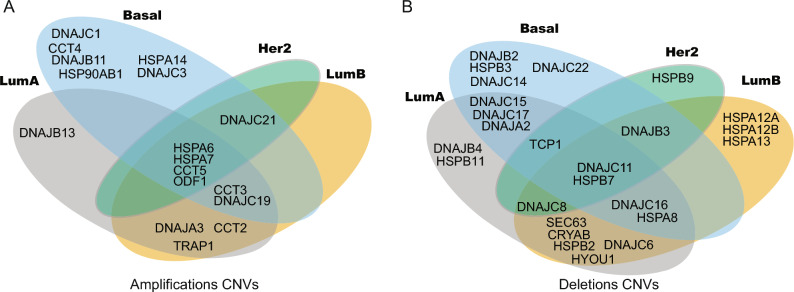
HSP CNV on BRCA distributed according to molecular subtypes (TCGA cohort). Venn’s diagrams showing deletions CNV (**A**) and amplifications CNV (**B**) obtained by GAIA (cut-off: amp-CNV ≥ 0.3; del-CNV ≤ − 0.3).

In the context of molecular subtypes of BRCA, we analyzed amplification CNV (Fig. 3). We found a group with four genes (HSPA6, HSPA7, CCT5, and ODF1) amplified in all molecular subtypes. The Basal subgroup exhibited the highest number of amp-CNV alterations, involving 13 HSP genes, and also presented the major number of subtype-specific amp-CNV (CCT4, DNAJB11, HSP90AB1, HSPA14, DNAJC1, and DNAJC3). In the case of luminal tumors, Luminal A showed 10 amp-CNV, with only DNAJB13 as subtype-specific alteration, and Luminal B presented 10 amp-CNV. Luminal A shared 3 amp-CNV with Luminal B (CCT2, TRAP1, and DNAJA3), and the alteration on CCT3 and DNAJC19 with Basal and Luminal B. HER2 subtypes showed a minor number of alterations (5), these include the common group to all subtypes and CNV on DNAJC21 shared with Luminal B and Basal. In the analysis of del-CNV alterations (Fig. 3), we found that DNAJC11 and HSPB7 genes with significative del-CNV across all molecular subtypes. The Luminal A subtype exhibits the highest number of del-CNV alterations present in 16 HSP. Notably, this includes subtype-specific CNV alterations in the genes HSPB11 and DNAJB4. Luminal B presented 14 del-CNV, including HSPA12A, HSPA12B, and HSPA13 as subtype-specific. Luminal A shared 5 del-CNV with Luminal B (DNAJC6, SEC63, CRYAB, HSPB2, and HYOU1), with Basal shared del-CNV on DNAJC15, DNAJC17, and DNAJA2. Also, Luminal A shared del-CNV on genes DNAJC16 and HSPA8 with Basal and Luminal B. The Basal subgroup presented 13 del-CNV, and also presented the major number (4) of subtype-specific del-CNV (DNAJB2, HSPB3, DNAJC22, and DNAJC14). HER2 subtype showed a minor number of del-CNV (6), these include the common group to all subtypes, the specific subtype del-CNV on HSPB9. HER2 shared CNV on DNAJB3 with Luminal B and Basal, and del-CNV alterations in TCP1 were shared with Luminal A and Basal.

**Figure 4:**
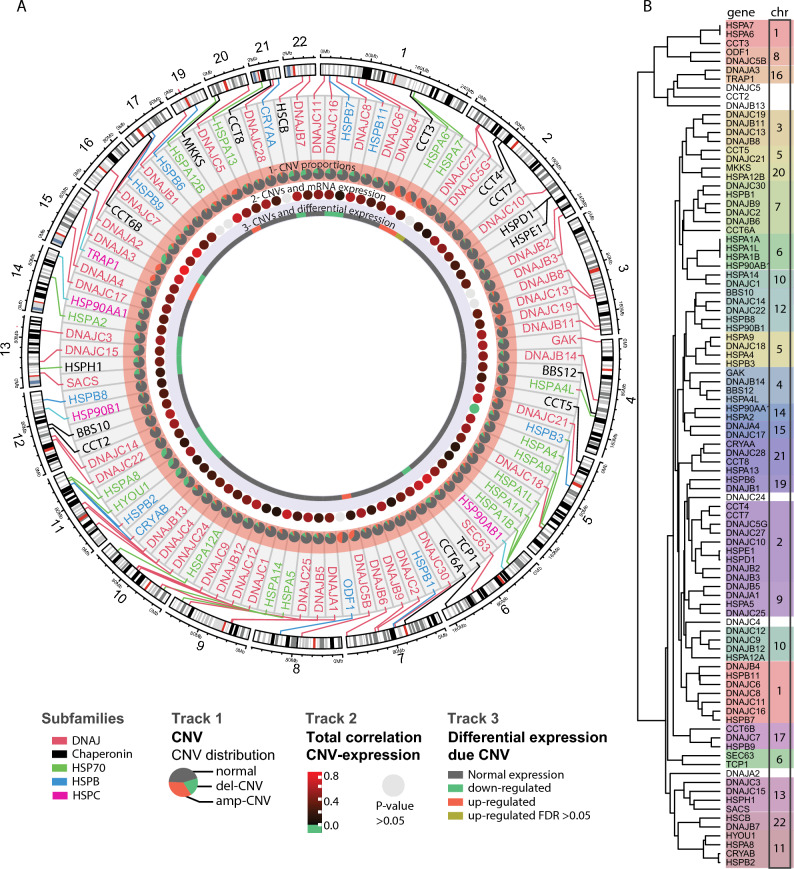
CNV of HSP genes in BRCA in the human genome context and mRNA expression. (**A**) Genomic localization of HSP genes. Track 1: Proportions (pies graphs) of CNV in TCGA, in green del-CNV (cut-off CNV value −0.3), and in red amp-CNV (cut-off CNV value 0.3). Track 2: Spearman correlation of CNV and mRNA of TCGA cohort. Track 3: differential gene expression due to CNV, genes with P-value > 0.05, and low-level expressed genes (1 CPM in at least 5 samples) were represented in gray. (**B**) Similarities analysis of CNV alterations on HSP genes. Dendrogram of HSP genes according to Euclidean distance.

We found that the frequency of CNV alterations varies between genes, our observation revealed that in 89 genes, the proportion of patients without any CNV alteration surpasses 60%. On the other side, there were HSP genes with more than 35% of the cohort presenting CNV alterations, this is the case of CCT3, HSPA6, HSPA7, DNAJC5B, and ODF1 with amp-CNV, and genes with del-CNV HSPB2, HYOU1, CRYAB, and DNAJA2 (Fig. 4A Track 1).

Finally, having thoroughly examined the CNV profiles within each HSP subfamily, and subsequently within the molecular subtypes of BRCA, we conducted a comprehensive assessment of these alterations in pursuit of identifying distinct and characteristic profiles. To achieve this goal, we performed clustering analysis to evaluate the similarity of the genes based on their CNV alterations. Upon examining the resulting dendrogram, we observed the emergence of small, distinct clusters, each comprising a range of 2 to 6 HSP genes, most of which are located near each other in genomic loci (Fig. 4B).

To evaluate the impact of CNV on the expression of the HSP family genes, as a first approach we analyzed the association of these two variables by correlating the number of copies of each HSP with the levels of mRNA in the TCGA BRCA cohort. We found that 26 HSPs presented positive correlation coefficients greater than 0.5. On the other hand, just one HSP presented a negative coefficient (-0.11) (Fig. 4A. Track 2). Furthermore, we utilized iGC to identify the HSP genes that exhibited differential expression as a result of CNV^19^. We analyzed only those genes that demonstrate either up-regulation or down-regulation in a minimum of 20% of the samples and performed a comparison between samples with and without the presence of CNV. As a result, we found 19 differentially expressed HSP genes, of which 6 (CCT3, HSPA6, DNAJA3, TRAP1, DNAJC5, and DNAJC5B) were up, and 13 (DNAJA2, TCP1, HSCB, HYOU1, DNAJC3, DNAJC16, HSPH1, DNAJC15, CRYAB, SACS, HSPB2, HSPB7, and HSPA8) were down (Fig. 4A. Track 3). Subsequently, we divided the patients according to the molecular subtype of BRCA and again related the CNV with the expression of the HSP (Fig. S2). The correlation coefficients were positive in most of the HSP, observing a similar pattern in the 4 subtypes, however, we found some HSP with distinctive correlation coefficients for some specific subtypes. Such is the case of some members of the chaperonin family, particularly the CCT-TRiC complex where the coefficients were lower in the HER2 molecular subtype (CCT4, CCT5, CCT7, and CCT8). On the other hand, we observed greater variability between the BRCA subtypes in HSP located on chromosome 2 (DNAJB3, DNAJB2, HSPE1, HSPD1, and DNAJC10), chromosome 3 (DNAJB11 and DNAJC19), chromosome 4 (GAK), chromosome 14 (HSP90AA1 and HSPA2) and chromosome 15 (DNAJA4 and DNAJC17).

### Clinical implications of copy number variations on HSP genes

To ascertain the clinical implications of CNV on HSP genes, we conducted additional evaluations to assess the proportional risk of the patients with CNV on every HSP gene. We identified that alterations in 19 genes modified the outcome of patients. Some genes show improved clinical outcomes when the number of copies of DNA regions increases, such as in the case of DNAJA1, HSPA12A, and HSPA2. On the contrary, the increase in copies on CCT4, CCT7, DNAJC13, DNAJC21, HSPA1L, HSPA1A, HSPA1B, SEC63, DNAJC5B, ODF1, HSPA5, DNAJC1, DNAJC14, DNAJA2, HSPA13, and CRYAA leads to a worse survival perspective (Table 1).

**Table 1:** Overall survival risk of patients with CNV alterations on HSP genes.

Gene	Coeff	P-val
CCT7	1.8843	0.0051
DNAJC14	1.5478	0.0187
DNAJC13	1.4637	0.0078
CRYAA	1.4090	0.0003
CCT4	1.3960	0.0091
HSPA1L	1.2020	0.0171
HSPA1A	1.2020	0.0171
HSPA1B	1.2020	0.0171
DNAJC21	1.1073	0.0160
HSPA5	0.9229	0.0137
DNAJC1	0.8586	0.0489
HSPA13	0.8406	0.0083
ODF1	0.7755	0.0017
DNAJC5B	0.6279	0.0103
SEC63	0.5755	0.0172
DNAJA2	0.4848	0.0360
DNAJA1	–0.9727	0.0364
HSPA2	–1.2275	0.0058
HSPA12A	–1.2581	0.0008

The continuous values of CNV on HSP genes were applied to the univariate Cox’s model. Only HSP genes with P-val < 0.05 are tabulated.

**Figure 5:**
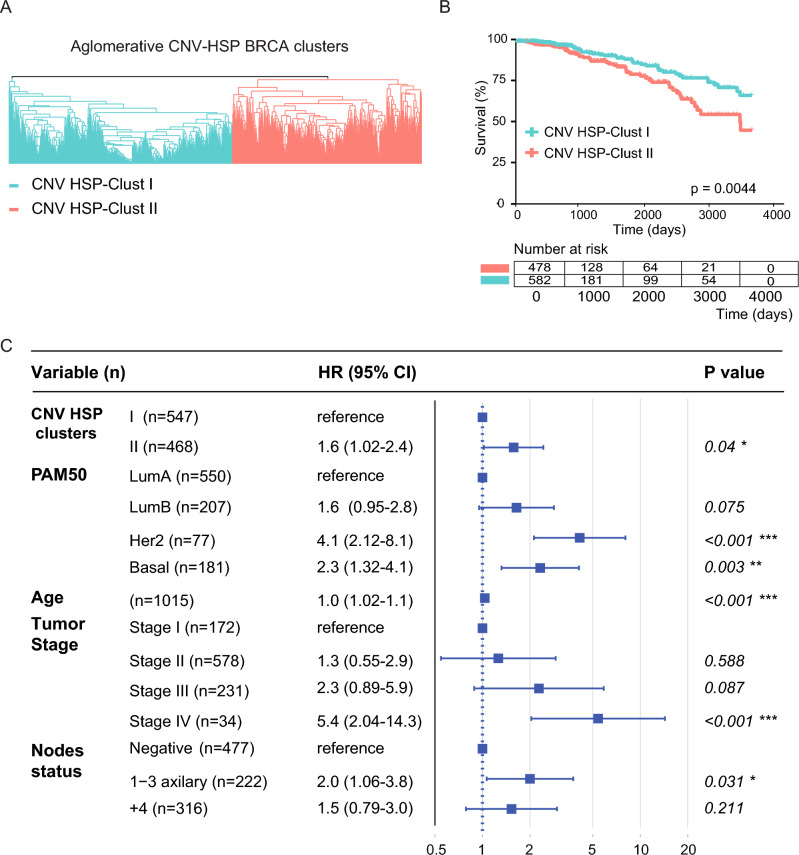
Clinical implications of patient’s groups obtained by the profile of CNV on HSP genes (TCGA cohort). (**A**) Dendrogram of patient groups obtained by a non-supervised hierarchical clustering algorithm. The partition of the two main groups was determined by the Silhouette coefficient^20^. (**B**) Survival graph over time using the Kaplan–Meier method to compare the survival curves of different CNV-HSP clusters. The p value of the log-rank test is also plotted. (**C**) Multivariate Cox’s model of CNV HSP clusters (CNV-HSP I as reference).

Next, we classified the TCGA cohort based on the similarities in the CNV profile presented in HSP genes. Through a non-supervised statistical method we divided the patients into two main groups denominated CNV HSP-Clust I and CNV HSP-Clust II respectively (Fig. 5A). We observed that CNV HSP-Clust II exhibited a higher prevalence of amp-CNV and del-CNV compared to CNV HSP-Clust I, with rates of 11.16% and 6.89% for amp-CNV, and 12.86% and 8.73% for del-CNV, respectively. We found significant differences (Wilcoxon test) in the amount of amp-CNV (p = 2.2e−16) and del-CNV (p = 2.177e−10) between both clusters. Subsequently, we performed a survival analysis between the two groups, and we observed that CNV HSP-Clust I had a better clinical outcome (log-rank test p = 0.0044) (Fig. 5B). To assess the relevance of CNV of HSP genes in the outcome of patients and the relation with other known clinical variables, we performed a multivariate Cox´s model. Our findings indicate that the model identified HER2 and Basal molecular subtypes, stage IV, 1–3 axillary nodes, and CNV HSP-Clust II as independent risk factors. Patients belonging to CNV HSP-Clust II present 1.6 more risks compared to CNV HSP-Clust I patients (Fig. 5C).

**Figure 6:**
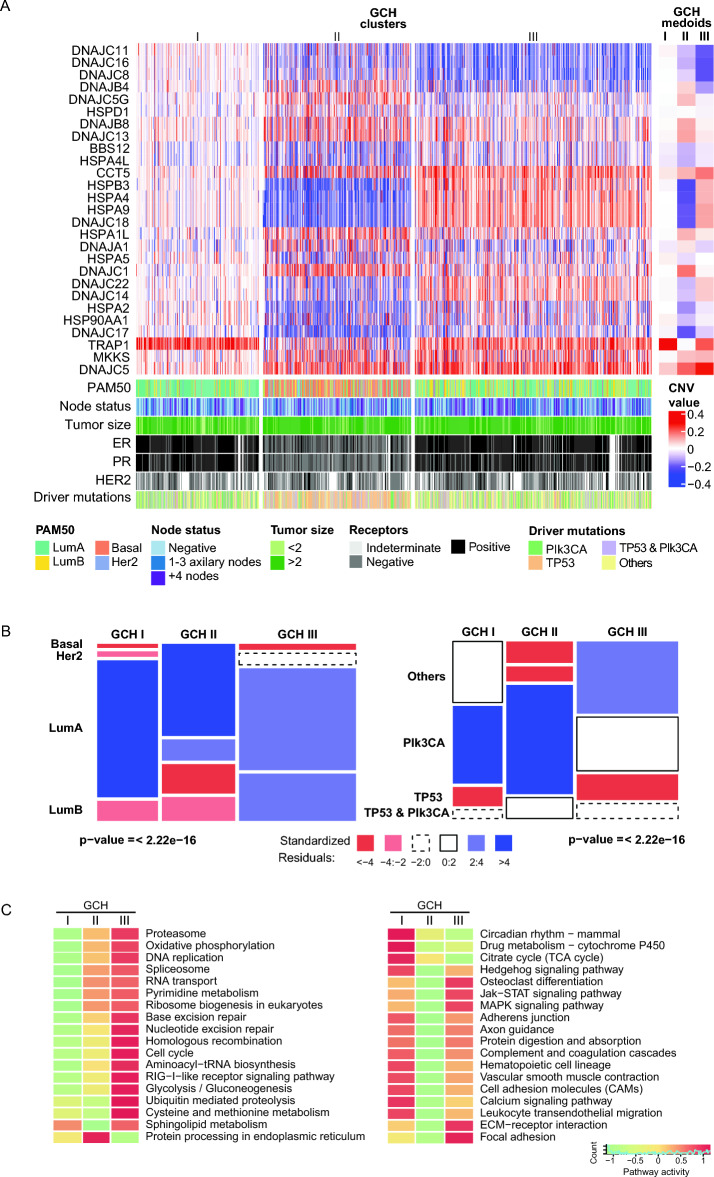
Galgo CNV-HSP subtypes (GCH) characterization. (**A**) Heatmap comparing CNV profiles within the Galgo CNV-HSP groups from the TCGA training set. Adjacent to the heatmap, we visualize the medoids for each HSP incorporated into our model. At the bottom of the heatmap, seven bars showing essential clinical variables in BRCA, including PAM50 molecular subtype, lymph node status, tumor size, IHC results for ER, progesterone receptor (PR), HER2 status, and the discerning driver mutations within our patient cohort. (**B**) Mosaic plot showing the composition and distribution of GCH groups relative to PAM50 subtypes and by driver mutations. For this, we performed a CHI-squared test and plotted the standardized residuals with a cutoff value of ± 2. The categories with standardized residuals far from zero, positive in blue, or negative in red, suggest an association between the variables. (**C**) Evaluation of altered molecular pathways in GCH subtypes, positively and negatively regulated gene sets from KEGG are plotted.

**Figure 7:**
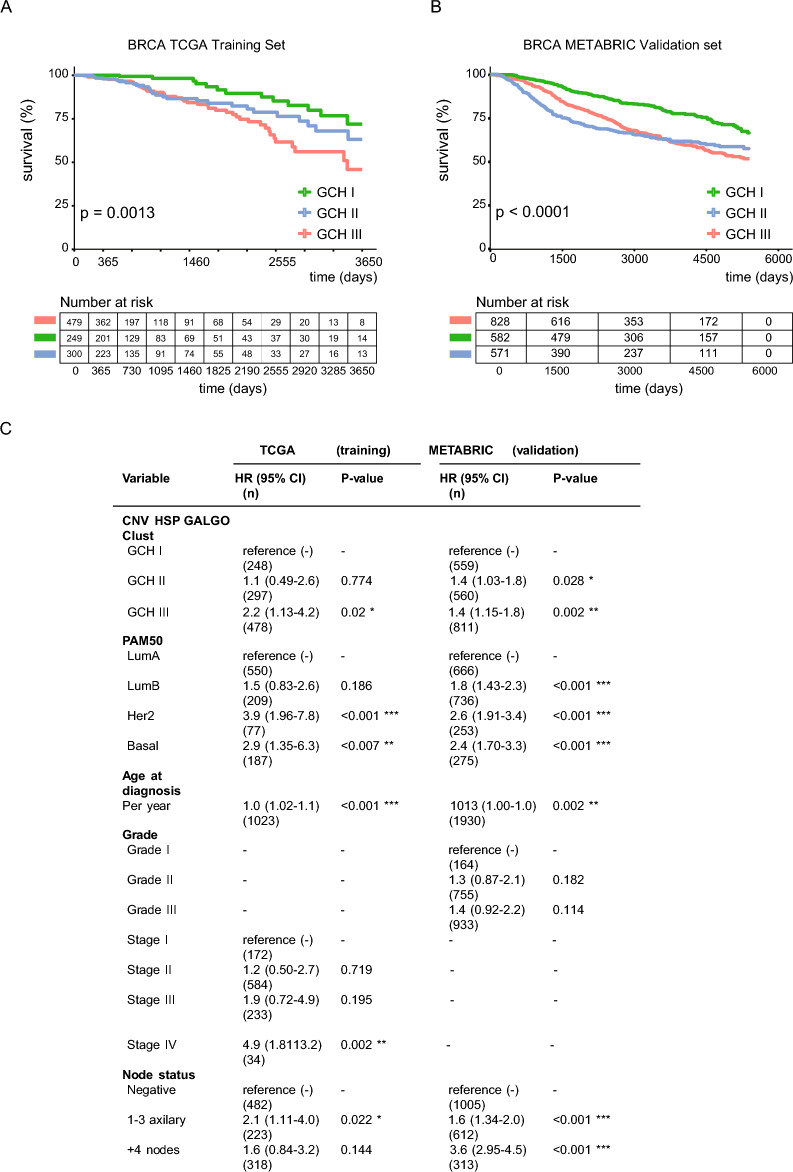
Evaluation of clinical characteristics of patients groups obtained from Galgo. Kaplan-Meier curves, comparing the overall survival between the Galgo CNV-HSP I, II, and III, in the training cohort (**A**) and the validation cohort (**B**). Statistical significance (p-value) was determined using the log-rank test. (**C**) Multivariate Cox model between Galgo groups, adjusted for classical BRCA predictors variables (Galgo CNV-HSP I as reference).

To further study the capability of CNV alterations of HSP genes as a better prognostic marker, we apply Galgo, an artificially intelligent genomic tool developed in our lab^21^. Galgo is a bi-objective algorithm oriented to finding cohesive groups with different clinical outcomes. We used TCGA cohort as a training set to run Galgo and then we validated the results in the METABRIC cohort. Briefly, with the patient groups determined by Galgo in TCGA, we get the CNV medoids values of selected HSP genes (Fig. 6A), and then we classify the METABRIC patients. Galgo selected 27 genes across subfamilies of HSP and split the cohort into three main groups: Galgo CNV-HSP I (GCH-I), Galgo CNV-HSP II (GCH-II), and Galgo CNV-HSP III (GCH-III) (Fig. 6A). The three groups have a non-independent distribution of patients relative to PAM50 subtypes (p < 2.22e−16) (Fig. 6B). The GCH-I cohort is specifically enriched in Luminal A cases to the detriment of the other subtypes. The GCH-II is the leading group with Basal patients and has more HER2 patients than the other groups. Meanwhile, GCH-III is enriched in Luminal A and B presenting a low number of Basal patients (Fig. 6B). About the distribution of known driver mutations in BRCA we found that TP53 is enriched in GCH II and is depleted in the other clusters. PIK3CA is the most frequent mutation found in GCH I, meanwhile, the category of other mutations (mutations on BRCA genes eg: GATA3, CDH1, PTEN, KMT2C, SPEN, and NCOR1) is the most prevalent in GCH III cluster (Fig. 6B). To assess the variations in the molecular pathways between the different GCH subtypes, we carried out a gene set enrichment analysis (GSEA), in which we identified significantly altered biological processes. To this end, we used the GAGE method^22^. Pathway information was sourced directly from the KEGG database (https://www.genome.jp/kegg/). KEGG facilitated the retrieval of 176 distinct gene sets, each corresponding to well-defined pathways^23^. From this analysis, we get the molecular pathways that were positively and negatively regulated for each of the GCH subtypes analyzed in the training cohort (Fig. 6C). The pathways related to DNA replication and repair, cell cycle, focal adhesion, and transendothelial migration of lymphocytes, among others, were among the most deregulated among the different GCH groups. For example, GCH I, which presented better prospects for patient survival (Fig. 7), showed down-regulation of pathways related to the cell cycle, DNA replication, and repair compared to the other GCH clusters. On the other hand, in GCH III we can observe a significant increase in activity in pathways related to the interaction of cell receptors with the extracellular matrix, which have an essential role in communication between cells, proliferation, adhesion, and migration, giving rise to more aggressive phenotypes, which is reflected in lower survival of the patients in this group (Fig. 7). Finally, we can also observe differences in the activity of pathways related to Jak-STAT and MAPK, which are involved in processes such as immunity, cell division, cell death, and tumor formation, supporting the differences in biological activity between the groups found.

The three GCH groups obtained by Galgo showed significant differences in overall survivals both in TCGA and METABRIC cohorts (Fig. 7A, B). We performed multivariate Cox’s model in both TCGA training set and METABRIC validation set (Fig. 7C). Both models are congruent in their results. In the most numerous METABRIC cohort both GCH-II and GCH-III groups are identified as independent covariates of other known variables, meanwhile, only GCH-III is a significant risk variable in TCGA set.

## Discussion

Breast cancer is one of the most studied neoplasms in the world, and many useful characteristics and classifications arise from the cellular, histological, and molecular criteria of this pathology. The HSP family actively participates in breast cancer, it has been demonstrated that the HSP expression profile is intimately related to clinical findings of patients’ outcomes^7,24^. Breast cancer typically exhibits a diverse array of genetic alterations, including both somatic mutations (acquired during a person’s lifetime) and germline mutations (inherited from parents). The DNA molecule can display these alterations in diverse manners, encompassing point mutations, insertions/deletions (indels), CNV, chromosomal rearrangements, and epigenetic changes. Copy number variation disorders arise from the dosage imbalance of one or more gene(s), resulting from deletions, duplications, or other genomic rearrangements that lead to the loss or gain of genetic material. The study of CNV has become increasingly important in understanding the genetic basis of complex traits and diseases and in the development of personalized medicine. In this work after careful analysis we evaluated DNA changes in HSP genes, the study denotes that point mutations (silent, missense, nonsense), indels, and alterations at translation start sites occur in HSP genes at low rates. It is worth noting that HSP are highly evolutionary conserved and ubiquitous in living organisms, they play a crucial role in the maintenance of cell homeostasis^25^. Insufficient observation of anomalous HSP genes may suggest a tendency to retain functional the molecular chaperone machinery. In further analyses, we determined that somatic CNV are the most frequent event in HSP genes of breast cancer cells. These alterations occur in elevated frequencies reaching in some cases 75% of the cohort. BRCA is a heterogeneous disease that can be classified into several molecular subtypes based on gene expression profiling. The four main subtypes of BRCA based on PAM50 gene expression profiling are Luminal A, Luminal B, HER2-enriched, and Basal-like. These subtypes differ in their clinical characteristics and response to treatment. Also, each subtype has different genomic characteristics^26^, this is reflected in the CNV of HSP, with the exception of a four genes group (HSPA6, HSPA7, CCT5, and ODF1) displaying amplifications across all molecular subtypes and the genes DNAJC11 and HSPB7 that showed copy-number loss in all subtypes, CNV in BRCA display different frequencies according to molecular subtypes. The Basal group exhibited the highest count of amp-CNV and also displayed the highest number of subtype-specific alterations encompassing both amplifications and deletions. In contrast, Luminal A is the group with the highest number of del-CNV. Interestingly, the luminal subtypes showed more del-CNV than amp-CNV contrary to Basal tumors. Notably, the HER2 subtype displayed the fewest CNV and featured only one subtype-specific deletion (HSPB9). As previously mentioned, the HSP family is divided into six defined subgroups, in many of which the members are closely linked, forming multimeric complexes at the protein level, acting as co-chaperones, and in some cases modulating the activity among them. After analyzing the CNV profiles, we did not observe any distinctive pattern in each subfamily; on the contrary, we observed high variability in the number and type of alterations. As observed in our findings, the primary factor influencing the magnitude of CNV alterations in HSP genes is their genomic location (Fig. 3B). Relative to the key importance of the alteration in the cancer process, we know that CNV comprises DNA segments ranging from 1 kilobase to megabases in length and these segments can contain one or several genes^27^. At this point, we can not determine if the CNV present in the HSP genes are really active participants in the cancerous phenotype or if this characteristic corresponds to other genetic elements contained in the same region. Future studies should be carried out to answer this question. Whatever the case, there is no doubt that these alterations can be used as phenotypic markers of cancer. Regarding the impact of CNV on the transcription process, we determined that overall CNV values typically correspond to shifts in transcript levels, although exceptions arise in cases of negative correlation like HSPB3. Some genes display a noteworthy positive correlation exceeding 0.5, reaching a peak of 0.82 with DNAJA2. However, the mechanisms governing this relationship remain undisclosed. Also, we observed that 20 CNV triggered differential expression of HSP genes, modulating seven up-genes (39% of the total amp-CNV), and 13 down-genes (59% of the del-CNV), suggesting that CNV are intimately related to mRNA expression mechanism. Extensive evidence has confirmed that the expression of HSP undergoes significant alterations in BRCA. Our research group conducted an integral transcriptomic study, comparing the expression HSP in tumor tissue samples against normal breast tissue samples, within the TCGA BRCA cohort^7^. This comprehensive analysis revealed that 24 members of this gene family displayed deregulation. Specifically, 13 genes exhibited elevated transcript levels, while 11 genes displayed a reduction in mRNA expression levels. During the present study, 19 CNV were found to instigate significant variations in the expression levels of HSP in BRCA samples. This raises the question of whether CNV may be the underlying cause of these expression disparities observed between normal and tumor tissues^7^. All six amplifications that led to a substantial increase in expression in tumor samples were also reported as up-regulated HSP when comparing normal and tumor tissues (CCT3, HSPA6, DNAJA3, TRAP1, DNAJC5, and DNAJC5B). Conversely, only four of the thirteen deletions that resulted in a significant decrease in expression in tumors were previously identified as down-regulated when comparing normal and tumor tissue (CRYAB, SACS, HSPB7, and HSPB2). However, it is important to acknowledge that the mechanisms governing expression levels are exceedingly diverse and complex. Additional studies are warranted to gain a deeper understanding of these events, as they encompass intricate processes with implications for our comprehension of cancer biology and therapeutics^9,28, 29^. Nowadays, the approach to BRCA management tends to be progressively individualized, several characteristics such as tumor size, nodal status, and patient age amongst others, are fundamentals to reach the accurate treatment. Also, genomics studies supporting precision medicine have evolved cancer diagnostics^2^. Molecular signatures are used to determine prognosis and treatment approaches, in BRCA most of these criteria have been developed mainly based on transcriptomic studies, such as the PAM50 intrinsic subtypes^3,30^, the 70-gene signature (MammaPrint)^31^, the signature of 21 genes (OncotypeDX)^32^ and EndoPredict^33^. Formerly, we postulate that the HSP expression profile is a prognostic factor for BRCA, but not with enough power to be independent of PAM50 molecular subtypes^7^. Here we demonstrated that the evaluation of CNV alterations on HSP genes could be helpful in dividing patients into groups with different clinical outcomes. Also, a further selection of CNV alterations by AI, creates a new signature independent of known factors, even of molecular subtypes based on PAM50. This permits the segregation of PAM50 subtypes (Luminal A, Luminal B, HER2, and Basal) into discrete subgroups with different prognoses. We arrive at these results by analyzing overall survival taking into account the CNV of all HSP genes and also using a reduced group of genes selected by the AI algorithm GALGO. This last was validated in a bigger step such as METABRIC cohort (Figs. 6 and 7C). It is known that many HSP participate in intrinsic cancer pathways contributing to cancer progression by promoting cell survival, inhibiting programmed cell death (apoptosis), enhancing drug resistance, and facilitating tumor growth and metastasis. Understanding the roles of HSP in BRCA is important for developing therapies and finding new prognostic markers. HSP have been explored as potential therapeutic targets in BRCA treatment, such as Hsp90 inhibitors, and have been investigated in preclinical and clinical studies as potential anticancer agents^34,35^. The deepest participation of HSP in the cellular pathways of cancer must be elucidated. Currently, there is a paucity of information regarding CNV within the HSP family in the context of BRCA. Furthermore, the regulatory mechanisms governing HSP expression in BRCA remain poorly understood, highlighting the need for additional complementary studies to elucidate these intricate dynamics. The exploration of CNV in this gene family holds the promise of yielding valuable insights into their contribution to tumor development. Such investigations not only enhance our comprehension of BRCA biology but also hold the potential for identifying novel prognostic markers and therapeutic targets, thus advancing our capabilities in the battle against breast cancer.

## Methods

### Data acquisition and preprocessing

To assess point mutations in HSP genes in BRCA, we obtained the data available from "The Cancer Genome Atlas" (TCGA—https://portal.gdc.cancer.gov/) through the R package "MAFTools"^36^. From this data, we examined the occurrence and nature of point mutations within each HSP gene, encompassing silent mutations, missense mutations, nonsense mutations, small insertions or deletions (indels) with or without reading frame shifts, as well as mutations at transcription start sites, across 986 primary breast tumor tissue samples.

To conduct CNV analysis of HSP genes, we employed data from two repositories: (1) initially, we acquired TCGA level 3 data (masked copy number) (https://portal.gdc.cancer.gov/) using the R package "TCGABiolinks"^37^, from 1094 primary breast tumor tissue samples. (2) Furthermore, we obtained CNV data from the METABRIC repository^38^ after authorization from the data access committee (DAC) at the "European Genome/Phenome Archive"—EGA (https://ega-archive.org/studies/EGAS00000000083). In this instance, CNV profiles were sourced from 1992 tumor samples. These data were programmatically downloaded by the pyEGA3 tool, using the Python programming language within a Linux environment. It is noteworthy that both repositories encompass data derived from the Affymetrix SNP 6.0 array platform. Pertinently, probes associated with variations in germ line copy numbers were excluded from our analysis. Consequently, our study meticulously focused solely on somatic copy number variations (masked-CNV). Subsequently, data from both the TCGA and METABRIC repositories underwent identical analytical pipelines.

This study has been approved by the Bioethical Committee of the Medical School of the National University of Cuyo, Mendoza, Argentina (0029963/2015). The experiments comply with the current laws of Argentina in which they were performed.

### CNV data preprocessing and analysis

After downloading the data, the CNTools R package from Bioconductor^39^ was used to process the segmentation data. For our study focused on the family of HSP genes, we identified the segments of interest based on the chromosomal location of these genes. Specifically, we selected the segments containing HSP genes and obtained a reduced segment matrix with the 95 HSP genes as rows and the samples as columns.

The R package GAIA (Genomic Analysis of Important Aberrations) was used to identify recurrent alterations in the BRCA cohort^15^. The GAIA package incorporates an iterative hypothesis testing approach, it uses conservative permutation tests to assess the significance of genomic alterations. By comparing the observed data with randomly permuted datasets, GAIA calculates p-values that are then corrected for multiple testing. This analysis was initially conducted on the entire BRCA cohort and subsequently performed on the different molecular subtypes of BRCA.

### Associations between CNVs and mRNA expression in the HSP gene family

To investigate the influence of CNV on the expression of HSP genes, we obtained the transcriptomic data (RNA-seq) of BRCA from the TCGA database. The data retrieval process was facilitated using the Bioconductor’s package TCGABiolinks^37^. Subsequently, we selected 1087 patients containing both expression and copy number data and related these values using the Spearman non-parametric test. This study was first carried out in the entire BRCA cohort and then in each of the BRCA molecular subtypes. On the other hand, we used the iGC package from Bioconductor, which allows an integrated analysis of gene expression and CNV^19^. Through this algorithm, we identified differentially expressed HSP due to CNV. To focus on deregulated genes in the general population, only genes showing CNV in at least 20% of the samples were analyzed.

### Clinical data acquisition and preprocessing

Clinical data and survival status updates for patients within the TCGA BRCA cohort were meticulously obtained through Bioconductor’s package TCGABiolinks^37^. To ensure the robustness of our patient selection, stringent inclusion criteria were applied, encompassing the availability of essential information such as lymph node status, tumor size, age, tumor stage, and PAM50 subtypes. This criteria resulted in the inclusion of 1097 patients. Exclusion criteria were thoughtfully employed, encompassing males, patients with indeterminate metastatic status at the time of diagnosis, and those belonging to the "Normal-like" subtype. Following this meticulous filtration process, 1060 patients remained, with 96 recorded events (deaths).

## Univariate Cox model

To investigate the association between CNVs in each HSP gene and patient survival, we applied the univariate Cox proportional hazards model. To validate the assumption of proportional hazards for the variables, we employed the Schoenfeld residuals method. This rigorous analysis was executed using the R package survival^40^.

## Survival analysis and multivariate Cox model

To assess differences in survival among the groups identified by the unsupervised algorithm, we computed survival functions and constructed Kaplan–Meier curves. Additionally, Log-Rank tests for survival comparisons were performed. We then refined our model by incorporating other conventional predictor variables in the context of BRCA, such as patient age, tumor stage, PAM50 molecular subtypes, and lymph node status, through the implementation of a multivariate Cox proportional hazards model. To ensure the validity of the proportional hazards assumption, we utilized the Schoenfield residuals method. All statistical analyses were conducted utilizing the R survival package^40^. In alignment with the principles of transparency and adherence to recommended standards, our survival analyses were conducted in accordance with the REMARK guidelines^41^. These guidelines provide essential recommendations for biomarker discovery and evaluation in the context of cancer research, ensuring the rigor and validity of our findings.

### GALGO artificial intelligence genomic tool

The tool "Galgo" developed in our laboratory is based on an elitist non-dominated sorting genetic algorithm ("Elitist Nondominated sorting Genetic Algorithm", NSGA-II), with a bi-objective approach, searching for groups with high support and cohesiveness, maximizing survival differences between these groups^21^. For the implementation of Galgo, we used two independent cohorts of BRCA patients: TCGA and METABRIC. The TCGA cohort was used as the training set. After training the models, we select the solutions with the highest C.Index for the partitions (k) of interest and use them to classify the samples of the test cohort, in this case, METABRIC, assigning each patient to one of the groups obtained by Galgo, according to the closest Pearson correlation distance to the medoids of each group.

### Characterization of patient’s clusters

To characterize the groups derived from Galgo, we established relationships between the Galgo groups with the PAM50 molecular subtypes, and subsequently, the prominent driver mutations documented in BRCA. Specifically, for the latter analysis, we partitioned the cohort into four distinct categories: TP53-positive patients, PI3K-positive patients, patients harboring both TP53 and PI3K mutations simultaneously, and a fourth group labeled "others", encompassing patients with mutations in driver genes such as GATA3, KMT2C, CDH1, SPEN, NCOR1, and PTEN. After stratifying the cohort based on these variables, we performed a Chi-squared test and evaluated the standardized residuals, applying a cutoff value of ± 2 for comparison. For PAM50 subtypes in METABRIC and TCGA, we used classification already published^7^.

Finally, to perform the Gene Set Enrichment Analysis (GSEA), we employed the R package GAGE, as detailed in^22^. In this analysis, groups created by Galgo were compared to the biological processes showing the most significant changes according to transcriptome profiles. In this case, we utilized gene sets sourced from the KEGG database (https://www.genome.jp/kegg).

### Supplementary Information

Supplementary Figures.

Supplementary Table S1.

## Data Availability

The TCGA datasets analyzed during the current study are available in the Genomic Data Commons Data Portal (National Cancer Institute, NIH, USA) repository (https://portal.gdc.cancer.gov/projects/TCGA-BRCA). The METABRIC data is available, after authorization from the data access committee (DAC), at the "European Genome/Phenome Archive"-EGA (https://ega-archive.org/studies/EGAS00000000083). Both METABRIC and TCGA offer anonymized data, and all experiments were conducted in accordance with relevant guidelines and regulations, explicit informed consent for the use of biological samples and associated clinical data for genomic analysis and research purposes was obtained from all subjects or their legal guardians. (https://www.cancer.gov/ccg/research/genome-sequencing/tcga/history/ethics-policies).
