# Supplementary material for: Mutational landscape of HSP family on human breast cancer
Source: Sci Rep. 2024 May 30;14:12471. doi: 10.1038/s41598-024-61807-8 (PMC11139924; doi:10.1038/s41598-024-61807-8)
Supplement: Supplementary file 1 — Supplementary Figures. [file 41598_2024_61807_MOESM1_ESM.pdf]

Supplementary Material

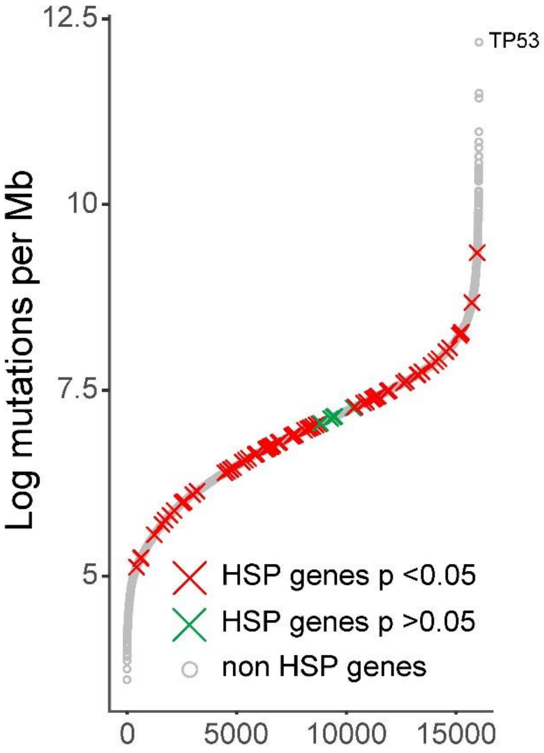

Figure S1. Comparision of mutation frequency between 16041 genes from BRCA TCGA cohort (n= 986). The number of mutations per gene was normalized against its transcript length and scaled to megabase, then ordered and plotted. Statistical significances (p-value) of HSP genes were determined using the Wilcoxon test.

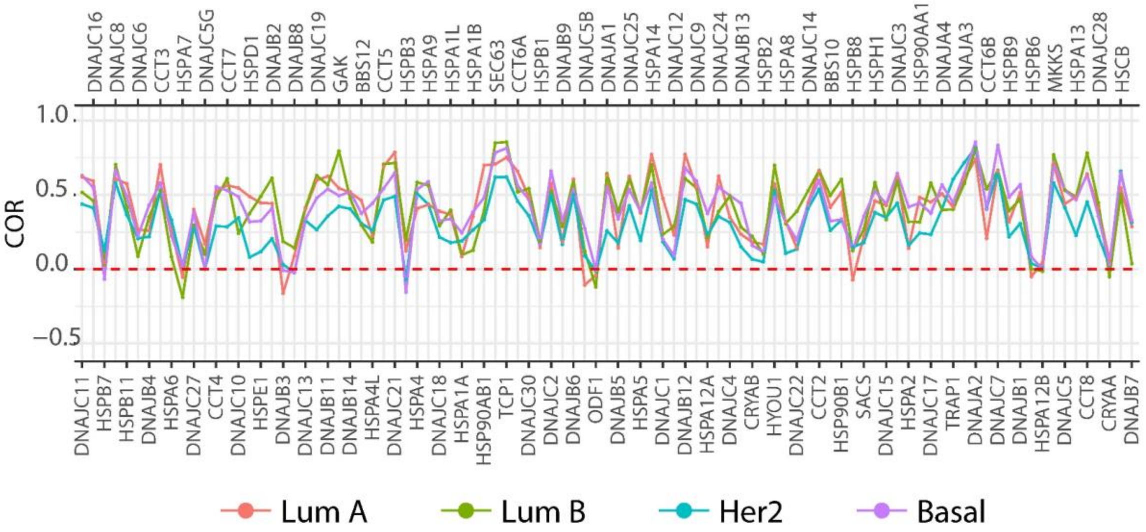

Figure S2. CNV and RNA expression of HSP genes according to PAM50 BRCA subtypes. Spearman correlation of CNV and RNA expression values in TCGA cohort divided by BRCA molecules subtypes.
